# Chronic arsenic exposure induces the time-dependent modulation of inflammation and immunosuppression in spleen

**DOI:** 10.1186/s13578-020-00448-6

**Published:** 2020-07-30

**Authors:** Nan Yan, Guowei Xu, Chenchen Zhang, Xuping Liu, Xin Li, Lin Sun, Da Wang, Xiaoxu Duan, Bing Li

**Affiliations:** 1 Environment and Non-Communicable Disease Research Center, Key Laboratory of Arsenic-Related Biological Effects and Prevention and Treatment in Liaoning Province, School of Public Health, China Medical University, No. 77 Puhe Road, Shenyang North New Area, Shenyang, 110122 Liaoning People’s Republic of China; 2 Department of Toxicology, School of Public Health, Shenyang Medical College, Shenyang, 110034 Liaoning China

**Keywords:** Arsenic, Inflammation, Immunosuppression, Spleen

## Abstract

**Background:**

Arsenic exposure has become a matter of worldwide concern, which is associated with immune-related diseases. However, little is known about its effect on inflammatory immune-related homeostasis. The purpose of our study was to understand the potential tuning of above responses exerted by chronic arsenic exposure.

**Methods:**

Kunming mice were treated with 25 and 50 mg/L sodium arsenite for 1, 3 and 12 months via drinking water. At different endpoints of arsenic exposure, all animals and the whole spleen of the mice were weighed. The total arsenic levels of spleen were determined by the HPLC-HG-AFS method. Splenic NF-κB, MAPK and NRF2 protein levels by treatment of 25 mg/L NaAsO_2_ for 1, 3 and 12 months and 25 mg/L and 50 mg/L NaAsO_2_ for 12 months were assessed by western blot. Total RNA of spleen was isolated and relative mRNA levels of *Foxp3*, *Il*-*10*, *Tnf*-*α*, *Il*-*6*, *Ifn*-*γ*, *Il*-*1β* and *Il*-*12* were measured by real-time PCR.

**Results:**

Our results shown that NF-κB were continuously activated with treatment of 25 mg/L arsenic from 1, 3 to 12 months and 50 mg/L arsenic for 12 months. The transcription factor *Foxp3* increased at 1 month but decreased at 3 and 12 months no matter 25 or 50 mg/L arsenic exposure. However, cytokine *Il*-*10* always showed increased trend in mice treated with 25 or 50 mg/L arsenic for 1, 3 and 12 months. The transcriptional profiles of *Tnf*-*α*, *Il*-*1β*, *Il*-*6*, *Ifn*-*γ* and *Il*-*12* revealed transient elevation at 1 and 3 months but shown significant decrease at 12 months on the whole. In addition, the sustained activation of inflammatory MAPK and anti-oxidative Nrf2 signaling pathways were observed in mice exposed to arsenic for 1, 3 and 12 months.

**Conclusion:**

In summary, our experiment in vivo suggested chronic arsenic exposure induces the time-dependent modulation of the inflammation and immunosuppression in spleen, which may be related to the activation of Tregs induced by MAPK/NF-κB as well as the increased transcription level of *Foxp3* and *Il*-*10*.

## Introduction

Arsenic contamination has become a global matter concerned. Studies have shown that arsenic exposure impedes cell growth and proliferation of immune function organs (spleen and thymus) [1–3]. A cohort study in Bangladesh has reported that exposure to arsenic during pregnancy caused the thymus gland shrank and its function impaired, leading to immunosuppression in childhood [4]. In addition, it has been demonstrated that arsenic could generate immunosuppressive responses by dose-dependent regulation of the NF-κB/Tregs/IL6/STAT3 signaling axis of mouse thymus cells [5]. It was found that immunosuppression in arsenic-exposed people in India is related to T cell proliferation and corresponding reduction of cytokines, such as TNF-α (tumor necrosis factor), IL-2, IL-4, IL-5, IL-10, etc. [6]. It is known that the expression of these immune inflammatory factors is inseparable from the involvement of NF-κB.

Duan et al. found that acute arsenic exposure activated MAPK/NF-κB signaling pathways in the thymus and spleen of mice, which ultimately stimulated the inflammation by the transcription and secretion of pro-inflammatory cytokines such as TNF-α, IL-1β and IL-6 etc. [7]. However, previous studies have failed to consider the effect of long-term exposure to arsenic in drinking water on inflammatory factors in vivo. Therefore, we are aimed to figure out the changes of inflammatory factors in vivo with the extension of arsenic exposure time.

A growing number of studies have shown that NF-κB maintains immune homeostasis through nonclassical pathways [5, 8]. It was found that NF-κB could exert immunosuppressive effect by Tregs (regulatory T cells) [8]. The abundance of Treg cells was increased in vitro culture of peripheral blood monocytes with arsenic of 0.1–1.0 μM [9]. In addition, it has been demonstrated that arsenic could generate immunosuppressive responses by dose-dependent regulation of the NF-κB/Tregs/IL6/STAT3 signaling axis of mouse thymus cells [5]. In animal model of experimental automine encephalopathy, arsenic (10 μg/L) resulted in an increase of Tregs but a decrease with arsenic (> 100 μg/L) in the spleen and peripheral blood [9].

The transcription factor Foxp3 is essential not only for the normal development of Tregs but also for their suppressive effects [10]. Studies have shown that the loss of Foxp3 expression over time impair the suppressive activity of Tregs [11, 12]. One of the mechanisms by which Tregs exert inhibitory activity is dependent on suppressive factors (IL-10, TGF-β, IL-35, etc.). So what is the effect of long-term exposure to arsenic on Foxp3 and IL-10, which is worth exploring.

Nrf2 signaling pathway is a classic antioxidant response element closely related to immunity [13]. Fry et al. reported that activation of NF-κB was associated with oxidative stress [14]. Therefore, the purpose of our research is to observe the MAPK/NF-κB mediated immune inflammatory and immunosuppressive responses in the spleen of mice under long-term arsenic exposure, and to provide a new treatment idea for long-term arsenic exposure.

## Materials and methods

### Reagents and chemicals

Sodium arsenite (NaAsO_2_, ≥ 99.0%) was purchased from Sigma Chemical Co. (St. Louis, MO, USA). NaAsO_2_ was dissolved in distilled water and diluted to the desired concentrations. Primary antibodies of NF-κB (C-20: sc-372), NRF2 (H-300: sc-13032), GSTO1/2 (FL-241: sc-98560), GCLC (H-300: sc-28965), and β-actin (1-19: sc-1616) were bought from Santa Curz Biotechnology (Santa Cruz, CA, USA); P-ERK (#9101), ERK (#9102), P-JNK (#9251), JNK (#9252), P-P38 (#9211), and P38 (#9212) were from Cell Signaling Technology (Cell Signaling, USA). The corresponding secondary antibodies were all purchased from Santa Curz Biotechnology (Santa Cruz, CA, USA). Real-time polymerase chain reaction (real-time PCR) kits were from Takara Co (Otsu, Japan). All other reagents were of the highest grade commercially available. Water used in all the preparations was distilled and deionized.

### Animals and experimental procedures

Ninety female Kunming mice (weighing 18–22 g, 6–7 weeks old) were obtained from the Center for Experimental Animals at China Medical University (Shenyang, China) with a National Animals Use License number of SCXK-LN2013-0007. All experiments and surgical procedures were approved by Animals Care and Use Committee at China Medical University, which complies with the National Institutes of Health Guide for the Care and Use of Laboratory Animals. All efforts were made to minimize the number of animals used and their suffering. The mice were acclimatized to standard laboratory conditions for 2 weeks before the experiments.

Mice were group-housed in stainless steel cage (10 mice per cage) in an air-conditioning room with temperature at 20 ± 2 °C, 12 h light: 12 h dark cycle, and year round relative humidity of 50–60%. All animals were allowed balanced food and drinking water ad libitum.

The desired concentrations of NaAsO_2_ (25 mg/L and 50 mg/L) were prepared freshly and provided to the experimental mice to drink ad libitum for 1, 3 and 12 months, respectively. Control mice were treated with deionized water parallelly. At the different endpoints of arsenic exposure, all animals were weighed and sacrificed after deep anesthetization. The whole spleen of the mice was quickly removed and weighed, immediately frozen in liquid nitrogen and stored at − 80 °C for future use.

### Determination of splenic arsenic levels

The dissected spleen of mice was washed with normal saline to remove blood, and then homogenized on ice with deionized water. Arsenic species, including arsenite (iAs^III^), arsenate (iAs^V^), monomethylarsonic (MMA) and dimethylarsinic (DMA) were measured by a high-performance liquid chromatography-hydride generation-atomic fluorescence spectrometer (HPLC-HG-AFS, SA-10 Atomic Fluorescence Species Analyzer, Titan, Beijing, China), consisting of a liquid chromatographic column, a hydride generation equipment, and an atomic fluoresce detector, as published in previous experiments [15]. Total arsenic (T-As) levels of spleen were calculated by summing up the levels of iAs^III^, iAs^V^, MMA and DMA in each sample. All samples were analyzed in triplicate, and the results were expressed as mean ± SD (n = 3).

### Total RNA isolation and real-time PCR analysis

Total RNA of spleen was collected for RNA extraction using a Trizol Reagent (Invitrogen, USA). Real-time PCR was conducted using a two-step method and an ABI 7500 real-time PCR system (ABI, USA). Then, equal amounts (500 ng) of RNA were used for reverse-transcription (RT) to cDNA synthesis by PrimeScript RT reagent kits with gDNA eraser (Perfect Real Time, Takara, Japan), and PCR amplification was performed by SYBR Premix Ex Taq II (Tli RNaseH Plus) kits (Perfect Real Time, Takara, Japan). The PCR step was performed with the thermal cycling conditions as follows: 1 cycle of initial denaturation (95 °C for 30 s), and 40 cycles of amplification (95 °C for 5 s and 60 °C for 34 s). Primers for mouse genes were designed by PRIMER 3 software and synthesized by Sangon Biological Engineering Technology (Shanghai, China) as provided in Table 1. Relative mRNA levels of different target genes were normalized to *Gapdh* and finally expressed as folds of control groups. Triplicate reactions were performed for each sample and the results are presented as mean ± SD (n = 4).

**Table 1:** Primer sequences used in real-time PCR analysis

cDNA	Primer sequences	Product length (bp)
Mus-*Foxp3* (NM_054039)	F:5′-CAGCTCTGCTGGCGAAAGTG-3′ R:5′-TCGTCTGAAGGCAGAGTCAGGA-3′	123
Mus-*Il*-*10* (NM-010548)	F:5′-GGGGCCAGTACAGCCGGGAA-3′ R:5′-CTGGCTGAAGGCAGTCCGCA-3′	92
Mus-*Ifn*-*γ* (NM-008337)	F:5′-AAGCGTCATTGAATCACACCTG-3′ R:5′-TGACCTCAAACTTGGCAATACTC-3′	92
Mus-*Il*-*1β* (NM_008361)	F:5′-TGCCACCTTTTGACAGTGATG-3′ R:5′-AAGGTCCACGGGAAAGACAC-3′	220
Mus-*Tnf*-*α* (NM_013693)	F:5′-CCTGTAGCCCACGTCGTAG-3′ R:5′-GGGAGTAGACAAGGTACAACCC-3′	148
Mus-*Il*-*12* (NM_001303244)	F:5′-TGGTTTGCCATCGTTTTGCTG-3′ R:5′-ACAGGTGAGGTTCACTGTTTCT-3′	123
Mus-*Il*-*6* (NM_031168)	F:5′-CTGCAAGAGACTTCCATCCAG-3′ R:5′-AGTGGTATAGACAGGTCTGTTGG-3′	131
Mus-*Gapdh* (NM_008084)	F:5′-TGTGTCCGTCGTGGATCTGA-3′ R:5′-TTGCTGTTGAAGTCGCAGGAG-3′	150

### Western blot analysis

Spleen protein was extracted for western blot using standard protocols. Briefly, total protein concentrations of spleen were determined by BCA reagent kit using bovine serum albumin (Santa Cruz, CA, USA) as the protein standard. An equal amount (30 μg) of protein was separated by 10% sodium dodecyl sulfate-polyacrylamide gel electrophoresis (SDS-PAGE), then transferred onto a PVDF membrane (Buckinghamshire, UK). The blotting membranes were saturated in blocking solutions (TBST, the Tris-buffered saline containing 0.1% Tween 20 and 5% skim milk) for 2 h at room temperature, followed by incubating with the following primary antibodies of NF-kB (1:2000), P-ERK (1:500), ERK (1:1000), P-JNK (1:500), JNK (1:500), P-P38 (1:500), P38 (1:1000), NRF2 (1:1000), GSTO1/2 (1:1000), GCLC (1:1000), β-actin (1:2000) overnight at 4 °C, respectively. On the 2nd day, membranes were washed with TBST and then incubated with corresponding secondary antibodies (1:2000–1:5000) for 2 h at room temperature. After that, membranes were incubated with chemiluminescence reagents (PicoWest Super Signal, Pierce Biotechnology, USA). The signals were visualized with electrophoresis gel imaging analysis system (MF-ChemiBIS 3.2, DNR Bio-Imaging System, Israel). The signals of β-actin (1:5000) were used to normalize protein loading. The results were representative of three mice of each treatment group.

### Statistical analysis

All the experiments were repeated three times to obtain the data and carried out at least in triplicate. All quantitative data were expressed as mean ± SD. The statistical significance among differences between experimental groups was determined by one-way analysis of variation (ANOVA) using least significant difference (LSD) method (SPSS 11.0, SPSS Inc., Chicago, IL, USA). *p *< 0.05 was considered to be statistically significant.

## Results

### The general status, body weight gain, splenic weight and T-As levels of the study mice

Mice were treated with different concentrations of NaAsO_2_ for 1, 3 and 12 months through drinking water. The amounts of water intake, food consumption, and the body weights were recorded every week. During the whole study period, all the mice showed normal activity, regular growth, and no other obvious abnormalities by visual inspection. We have not found any significant differences in body weight gain, suggesting no obvious growth inhibition happened among different experimental groups. Organ index is a common used index in toxicological evaluation, since the changes of a certain organ index could reflect some toxic effects (such as edema or atrophy) of a specific toxicant. In our experiments, the splenic index decreased with the growth of the mice, however, we found no obvious changes of the size and the weight of the mice spleen, nor any other visible changes at autopsy, among the control and the arsenic treatment groups within the same durations (1, 3 and 12 months). Meanwhile, we also carried out splenic histomorphology of each treatment group, whose results did not show obvious pathological changes, which was consistent with our observations of splenic weight or splenic index. So, the corresponding pathological results were not listed in this section. The splenic T-As levels of the study mice were determined and listed in Table 2.

**Table 2:** The body weight gain, splenic weight, and the concentrations of total arsenic (T-As, ng As/g tissue) in spleen of control and different experimental mice

Experimental groups	Duration (month)	Dose (mg/L)	Body weight gain (g)	Splenic weight (g)	T-As in spleen (ng/g)
Group 1	1	0	11.64 ± 1.83	0.11 ± 0.03	< LD
Group 2	1	25	11.98 ± 1.74	0.10 ± 0.03	31.46 ± 9.18
Group 3	1	50	11.76 ± 2.30	0.10 ± 0.02	39.95 ± 3.86
Group 4	3	0	19.61 ± 1.95	0.10 ± 0.03	< LD
Group 5	3	25	18.57 ± 3.86	0.10 ± 0.02	38.31 ± 5.87
Group 6	3	50	18.41 ± 1.27	0.10 ± 0.02	36.34 ± 19.82
Group 7	12	0	24.51 ± 3.59	0.10 ± 0.02	< LD
Group 8	12	25	23.21 ± 4.78	0.11 ± 0.02	47.80 ± 15.90
Group 9	12	50	24.80 ± 2.11	0.10 ± 0.03	52.42 ± 3.29

Mice were treated with 25 mg/L and 50 mg/L NaAsO_2_ for 1, 3 and 12 months through drinking water ad libitum, and the body weight of the mice was recorded every week. At the treatment endpoints, the entire spleen was removed and weighed (mean ± SD, n = 10). The total arsenic (T-As) levels of spleen were determined by the HPLC-HG-AFS method. Results were expressed as mean ± SD (n = 3). The limit of detection (LD) for T-As was 1 μg/L

* *p* < 0.05 compared with corresponding control mice

### Chronic arsenic exposure leads to NF-κB activation as well as regulatory T cells specification in spleen

**Fig. 1:**
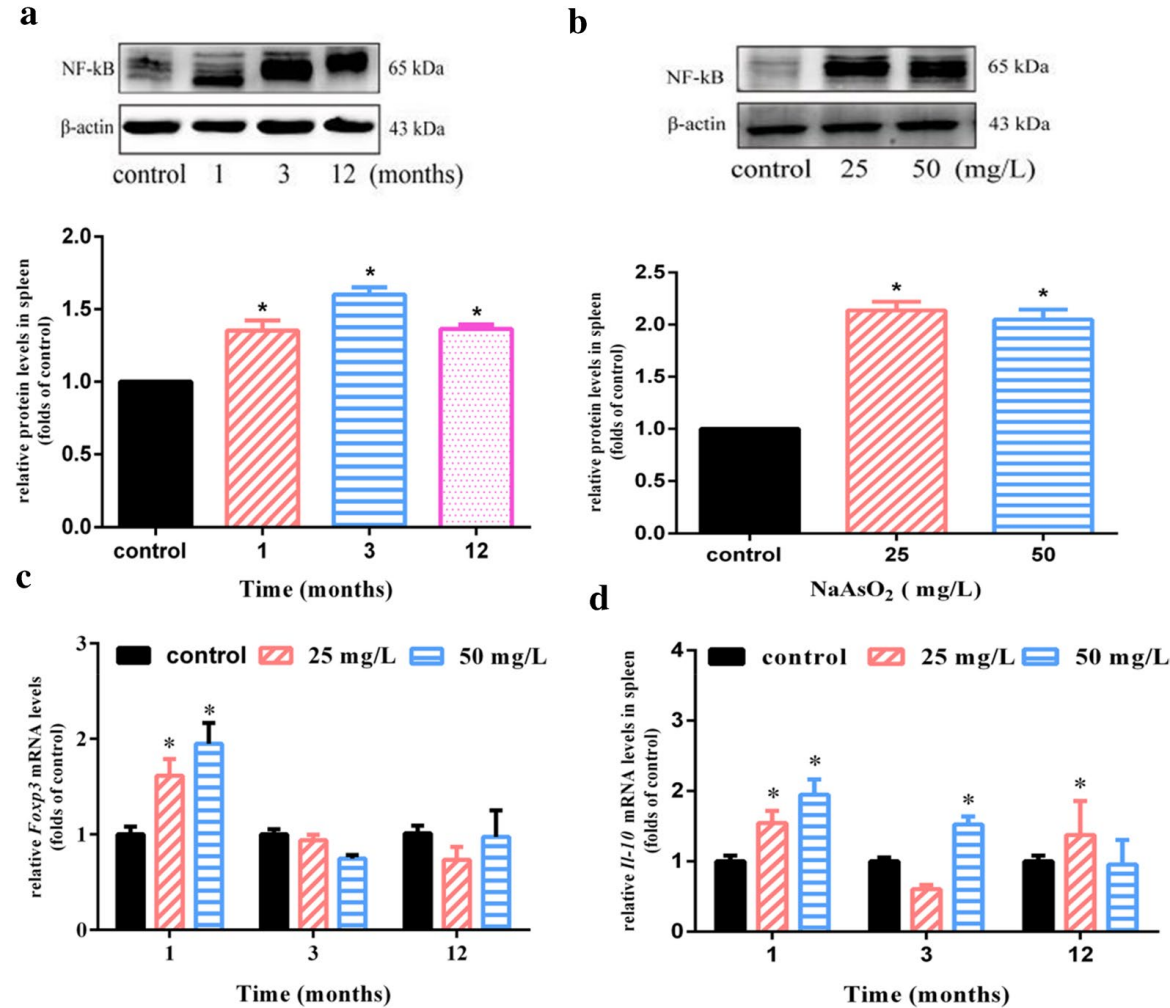
Chronic arsenic exposure leads to NF-κB activation as well as regulatory T cells specification in spleen. Mice were treated with NaAsO_2_ (25 mg/L and 50 mg/L) for 1, 3 and 12 months through drinking water ad libitum. Splenic NF-kB protein levels by treatment of **a** 25 mg/L NaAsO_2_ for 1, 3 and 12 months and **b** 25 mg/L and 50 mg/L NaAsO_2_ for 12 months were assessed by western blot. β-actin was blotted as the internal control. The exposure duration of the control group was 12 months in **a** and **b**. The density quantification of each band was presented as mean ± SD (n = 3). Total RNA of spleen was isolated and relative mRNA levels of *Foxp3* (**c**) and *Il*-*10* (**d**) were normalized to *Gapdh* and finally expressed as folds of control by real-time PCR. Results were expressed as mean ± SD (n = 4), and two such independent experiments were carried out. *p < 0.05 compared with corresponding control mice

On the one hand, NF-κB is widely involved in the initiation and development of inflammation [7, 16, 17]. Our results found that the levels of NF-κB protein expression in spleens of mice treated with NaAsO_2_ (25 mg/L) for 1, 3 and 12 months were significantly increased than those in the control group (Fig. 1a). Similarly, NF-κB was evidently activated in spleen of mice exposed to 25 and 50 mg/L NaAsO_2_ for 12 months, respectively (Fig. 1b). Those suggested that the expression of NF-κB was not affected by the duration and dose of arsenic treatment.

On the other hand, a study in vitro found that arsenic-induced NF-κB/IL-6 axis triggered immune responses by regulating the proliferation and differentiation of Tregs [5]. In our study, we observed an obvious increase in the transcription level of *Foxp3* (a transcription factor of Tregs) in the spleen of mice after 1 month of treatment with 25 and 50 mg/L NaAsO_2_ (Fig. 1c), but with the increase of treatment time (such as 3 and 12 months), this kind of increasing trend disappeared (Fig. 1c). As the main cytokine derived from Tregs, IL-10 also showed a rise in transcription level in groups treated with 25 mg/L NaAsO_2_ for 1 and 12 months as well as 50 mg/L NaAsO_2_ for 1 and 3 months compared with the control group (Fig. 1d). However, IL-10 showed no signs of increased transcription level in mice exposed to 25 mg/L NaAsO_2_ for 3 months as well as 50 mg/L NaAsO_2_ for 12 months (Fig. 1d). Taken together, these results suggested that long-term arsenic exposure induced the persistent activation of NF-κB as well as Tregs specification in a time-dependent manner.

### Chronic arsenic exposure affects cytokine profiles in spleen

**Fig. 2:**
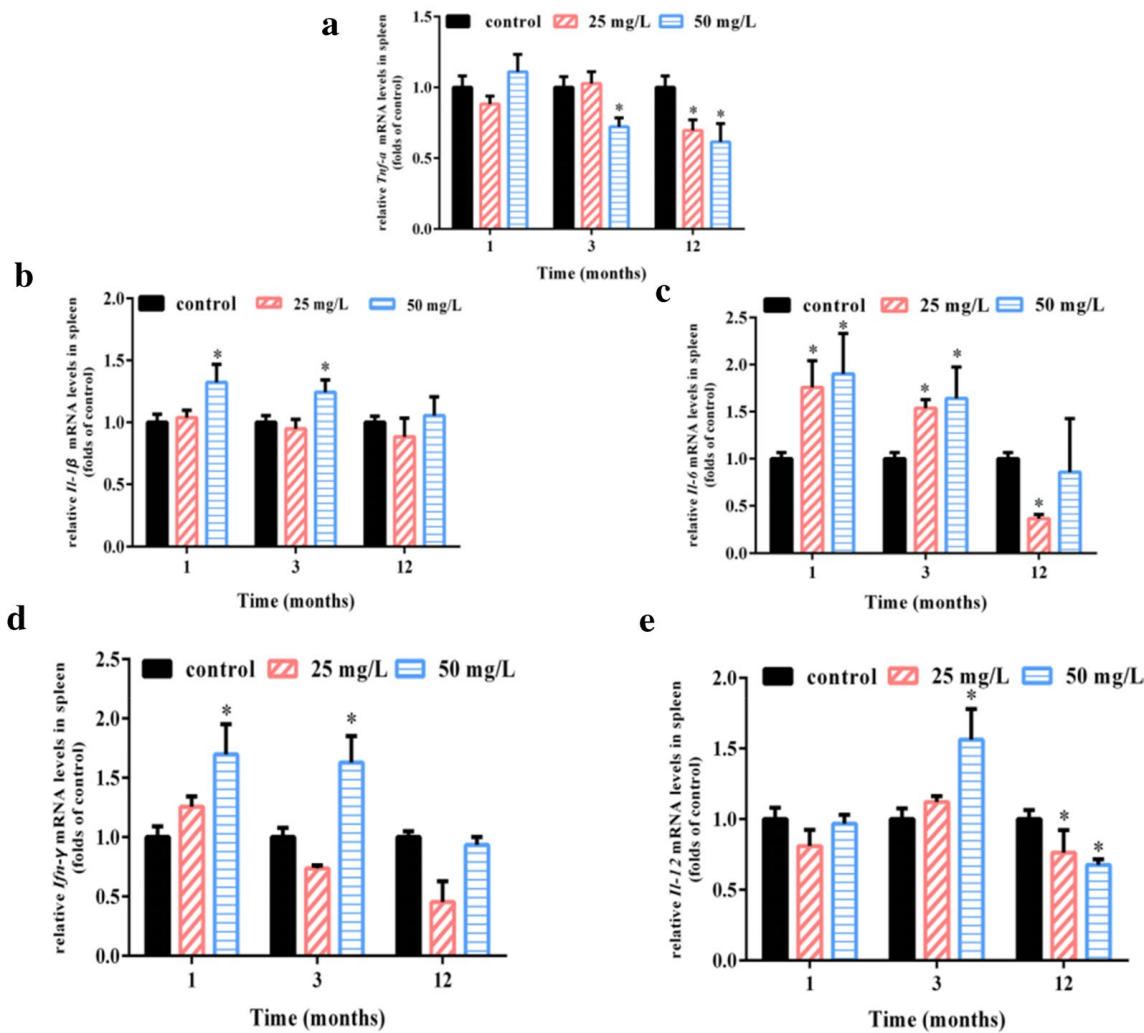
Chronic arsenic exposure affects cytokine profiles in spleen. Mice were treated with NaAsO_2_ (25 mg/L and 50 mg/L) for 1, 3 and 12 months through drinking water ad libitum. Total RNA of spleen was isolated and relative mRNA levels of *Tnf*-*α* (**a**), *Il*-*1β* (**b**), *Il*-*6* (**c**), *Ifn*-*γ* (**d**) and *Il*-*12* (**e**) were normalized to *Gapdh* and finally expressed as folds of control by real-time PCR. Results were expressed as mean ± SD (n = 4). *p < 0.05 compared with corresponding control mice

The disruption of arsenic on the immune inflammatory homeostasis was inseparable from the involvement of cytokines controlled by various immune cells [18–20]. Our research found that the classic inflammatory factor, *Tnf*-*α*, did not change at 1 and 3 months but declined at 12 months under 25 mg/L NaAsO_2_ treatment as well as decreased at 3 and 12 months in the case of 50 mg/L NaAsO_2_ exposure in mice (Fig. 2a). *Il*-*1β*, another inflammatory cytokine, remained unchanged after exposure to 25 mg/L NaAsO_2_ for 1, 3 and 12 months as well as 50 mg/L NaAsO_2_ for 12 months in mice spleen. However, increased transcription level of *Il*-*1β* occurred in mice exposed to 50 mg/L NaAsO_2_ for 1 and 3 months (Fig. 2b). *Il*-*6*, as a prominent pro-inflammatory cytokine, showed dramatic increase in the mice treated with 25 and 50 mg/L arsenic for 1 and 3 months but decreased with 25 mg/L arsenic for 12 months (Fig. 2c). *Ifn*-*γ*, a hallmark cytokine of Th1 cells, is widely involved in cellular immunity. We have detected a significant transcription increase in mice exposed to 50 mg/L arsenic for 1 and 3 months meanwhile no obvious change was observed in the other exposure methods (Fig. 2d). *Il*-*12* is also an important inflammatory factor. In our study, the transcription level of *Il*-*12* increased at 3 months but decreased after 12 months of 50 mg/L arsenic exposure as well as decreased at 12 months of 25 mg/L arsenic (Fig. 2e).

Taken together, these results suggest that chronic arsenic exposure caused an immune-inflammatory imbalance in mice spleen.

### Chronic arsenic exposure activates MAPK pathway in spleen

**Fig. 3:**
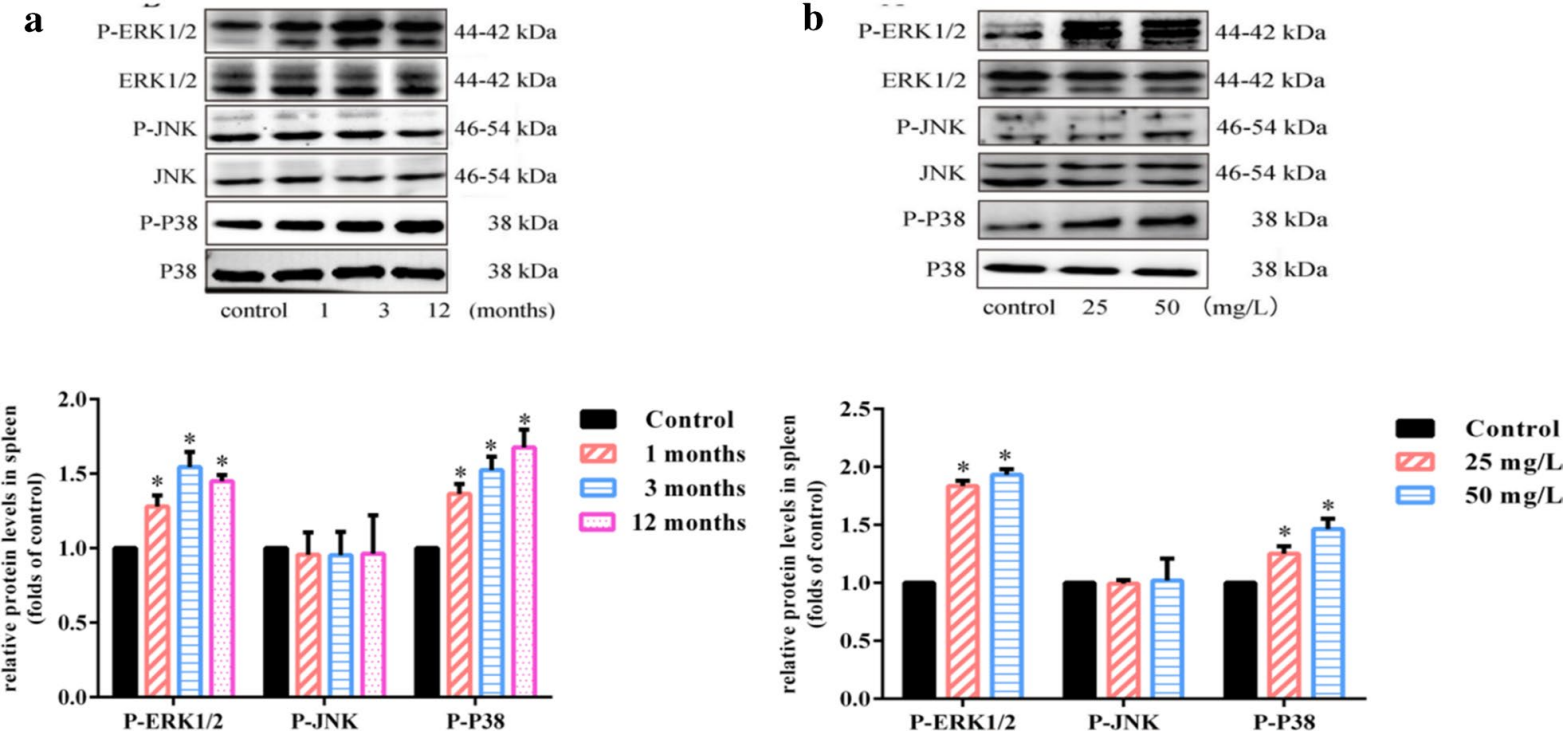
Chronic arsenic exposure activates MAPK pathway in spleen. Mice were treated with NaAsO_2_ (25 mg/L and 50 mg/L) for 1, 3 and 12 months through drinking water ad libitum. Splenic MAPK signaling pathway related protein levels by treatment of **a** 25 mg/L NaAsO_2_ for 1, 3 and 12 months and **b** 25 mg/L and 50 mg/L NaAsO_2_ for 12 months were assessed by western blot. β-actin was blotted as the internal control. The exposure duration of the control group was 12 months in **a** and **b**. The density quantification of each band was presented as mean ± SD (n = 3)

As the upstream of NF-κB, MAPK was found to play an important regulatory part in immune imbalance [21]. We observed the phosphorylation levels of ERK and P38 not JNK were obviously enhanced in mice treated with 25 mg/L NaAsO_2_ for 1, 3 and 12 months (Fig. 3a) as well as 50 mg/L NaAsO_2_ for 12 months compared with the control group (Fig. 3b). In summary, these indicated that long-term arsenic exposure could activate the MAPK signaling pathway in a time and dose-independent manner.

### Chronic arsenic exposure up-regulates nuclear factor NRF2 and its downstream targets in spleen

**Fig. 4:**
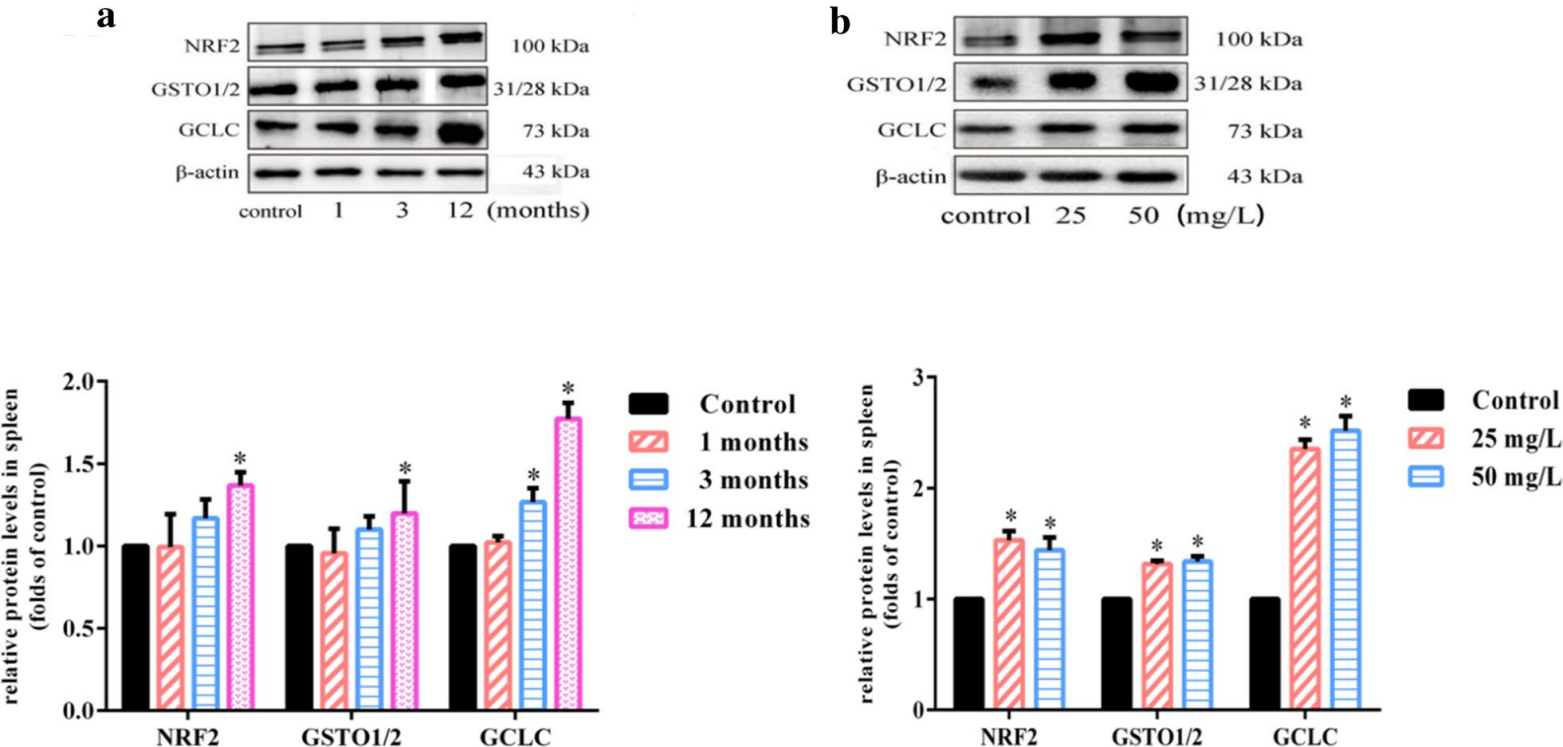
Chronic arsenic exposure up-regulates nuclear factor NRF2 and its downstream targets in spleen. Mice were treated with NaAsO_2_ (25 mg/L and 50 mg/L) for 1, 3 and 12 months through drinking water ad libitum. Splenic NRF, GSTO1/2 and GCLC protein levels by treatment of **a** 25 mg/L NaAsO_2_ for 1, 3 and 12 months and **b** 25 mg/L and 50 mg/L NaAsO_2_ for 12 months were assessed by western blot. β-actin was blotted as the internal control. The exposure duration of the control group was 12 months in **a** and **b**. The density quantification of each band was presented as mean ± SD (n = 3)

As a classical transcription factor for stress antioxidant and adaptive cellular defense response, NRF2 pathway has also been reported to play a regulatory role in immune imbalance [22]. Our results showed that protein expression levels of NRF2 and its downstream proteins GSTO and GCLC increased after 25 and 50 mg/L NaAsO_2_ exposure for 12 months (Fig. 4b) as well as 25 mg/L NaAsO_2_ for 3 months (Fig. 4a). This demonstrated arsenic largely up-regulated NRF2 and its downstream targets in mice spleen involved in maintaining homeostasis.

## Discussion

The immune homeostasis is essential for maintaining body health. The organ index is usually calculated by the ratio of organ weight to the whole-body weight, so the weights and indexes of immune organs are generally used to evaluate the body immune functions [23]. In our experiments, the splenic index decreased with the growth of the mice, however, no significant change in splenic weight was observed in all treatment groups, which was consistent with our observations of histomorphology. Totally, the current mice model did not initiate serious toxicological abnormalities. Therefore, we were aimed to explore the time-dependent modulation of the inflammation and immunosuppression of spleen by long-term arsenic exposure in vivo.

At present, the effect of arsenic on it is currently controversial. Although most studies believe that arsenic has immunosuppressive effects [24–26], there are also studies that suggest arsenic could be used as an immune stimulant to induce allergic reactions and autoimmune diseases [5, 27–29]. Immune regulation is necessary to control the occurrence and development of inflammation and autoimmune diseases. In all mechanisms of arsenic-induced regulatory dysfunction of immunity, the activation of NF-κB is inseparable.

On the one hand, NF-κB is widely involved in the immune inflammatory response caused by arsenic. Duan et al. found that acute arsenic exposure induced NF-κB activation and then stimulated the generation of inflammatory factors to induce the development of inflammation in the spleen and thymus of mice [7]. Arsenic could induce inflammation, which also was occurred in humans [14], rats [30], dendritic cells [18, 31] and avian brain tissue [32], etc. As shown in our study, we found that the spleen continuous NF-κB of arsenic-exposed mice, that was, it did not change with the extension of the exposure time (even for 12 months). This was not observed in previous studies. The transcription levels of various immune cytokines regulated by NF-κB also increased, such as the classic inflammatory factor clusters (TNF-α, IL-1β and IL-6) as well as non-classical immune inflammatory factors (IFN-γ and IL-12). The increase in expression of NF-κB as well as controlled immune-inflammatory factors of it with arsenic treatment suggested that chronic arsenic exposure induced the initiation and development of inflammation as acute arsenic exposure [7]. However, unlike acute arsenic exposure, with the increase of arsenic exposure time during the long-term arsenic exposure, the increasing trend of these immune inflammatory factors gradually disappeared, and even a decline phenomenon appeared at 12 months, which may be attributed to the alternative immune regulation mechanism of NF-κB.

On the other hand, NF-κB (especially the subunits c-rel and p65 in the classical pathway) is necessary for the development and function of Tregs [33]. Recent studies have found that the P65 subunit can maintain the abundance of regulatory cells and prevent the occurrence of autoimmune diseases [34, 35]. Levels of Tregs in mice deficient in NF-κB decreased significantly, and NF-κB was also shown to up-regulate Foxp3 expression [36]. Foxp3 cannot be used as a unique symbol of human Treg cells [37]. But for all Tregs, Foxp3 is nevertheless an important gene to regulate development and function of them. Its constitutive expression is the decisive factor driving the immunosuppressive function of mouse and human Tregs [38]. In mice, for instance, the Foxp3 gene mutation led to severe systemic autoimmune inflammatory diseases (Scurfy disease) [39]. Our results showed that the transcription level of Foxp3 in the spleen of mice was increased in the early stage (such as 1 month) of arsenic exposure, and in the middle and late stages (such as 3 and 12 months) of exposure, this trend no longer appeared, which suggested that the early stage of chronic arsenic exposure may induce the production of immunosuppression in the spleen of mice. IL-10, as the main anti-inflammatory cytokine and immunosuppressive factor produced by regulatory T cells, also showed a significant increase in the early stage of arsenic treatment. This result further supported the induction of immunosuppression in the spleen of mice at the beginning of chronic arsenic exposure.

Choudhury et al. found that arsenic triggered dose-dependent and differential immune responses in thymocytes by regulating the NF-κB/IL-6 axis, that was, high concentration of arsenic produced immunosuppression marked by high expression of IL-10 and Foxp3 as well as the inflammatory response marked by increase of TNF-α, IL-1β and IL-6 under low level arsenic [5]. Taken together, it indicated that the immunosuppression induced by arsenic was dose-dependent as well as the change of immune-inflammatory factors was involved in this process. We found that immunosuppression by arsenic may be time-dependent and it is important that immune inflammatory factors played a vital role in this process. It has been demonstrated that stress signals elicited by pro-inflammatory cytokines lead to the degradation of Foxp3 through the action of the E3 ubiquitin [40]. For instance, IL-1β down-regulated TGF-β-induced Foxp3 expression [41] and TNF-α activated protein phosphatase1 dephosphorylation of the Ser418 site of Foxp3 [42], both possibly receding suppressive function of Treg cells. In addition, IL-6 restrains Treg differentiation, which is an important mechanism involved in the pathogenesis of SLE [41]. Therefore, in our study, we have reasons to believe that the decrease in Foxp3 expression during mid-term arsenic exposure may be due to increased transcription levels of inflammatory factors. In fact, IL-10 inhibits the release of pro-inflammatory mediators from monocytes/macrophages and therefore inhibits the LPS and IFN-γ-induced production of TNF-α, IL-1β and IL-6 [43]. IL-10 is a rather late cytokine being produced after the pro-inflammatory mediators. Upon CD4^+^ T cells were activated in vitro, the presence of IL-10 causes these cells to develop a regulatory phenotype. In this manner, I type regulatory T cells (Tr1 cells) arise that secrete IL-10 but not Foxp3 and suppress antigen-specific effector T cell responses via a cytokine-dependent mechanism [44, 45]. Our results suggested that the inconsistent rise in the levels of IL-10 and Foxp3 in the middle and late stages of arsenic treatment (3 and 12 months) may be due to the role of Tr1 cells, which of course requires further researches to confirm this hypothesis. The continuous increase of IL-10 in the later stage (such as 12 months) exerts its anti-inflammatory and immunosuppressive effects by inhibiting the expression of various inflammatory cytokines, which also explains the phenomenon of the decline in the transcription level of immune inflammatory factors.

As an upstream signal of NF-κB, MAPK is widely involved in the arsenic-induced immune inflammatory process. The continuous activation of MAPK in our study was beneficial to induce the formation of various immune inflammatory factors. In addition to the classic antioxidant effect of the nrf2 signaling pathway, the specific deletion of Foxp3 in Nrf2 activated mice caused the loss of immune tolerance in mice, suggesting that nrf2 signaling is involved in the regulation of immune homeostasis [46]. Nrf2 deficiency has been shown to trigger an autoimmune inflammatory response in multiple organs [47, 48]. These results can also explain the decline of inflammatory cytokines in the spleen of mice in the late stage of arsenic exposure.

## Conclusions

In summary, our research found that long-term chronic arsenic exposure will break the body’s immune dynamic balance, which mainly shows the inflammatory response at the initial stage of exposure as well as the immunosuppression at the later stage of treatment. This will provide a new idea for the prevention and treatment of chronic arsenic poisoning in the future.

## Data Availability

All data are fully available without restriction.

## Abbreviations

TNF-α: Tumor necrosis factor
Tregs: Regulatory T cells
Tr1 cells: I type regulatory T cells
